# REALITIES in health disparities: Researching Evidence-based Alternatives in Living, Imaginative, Traumatised, Integrated, Embodied Systems

**DOI:** 10.3389/fpubh.2024.1391084

**Published:** 2024-06-19

**Authors:** Marisa de Andrade, and The REALITIES Consortium

**Affiliations:** School of Health in Social Science, University of Edinburgh, Edinburgh, United Kingdom

**Keywords:** research-practice-policy, public health, complex systems, inequalities, community assets, health disparities, arts-informed approaches, participatory action research

## Abstract

**Introduction:**

Under the backdrop of pervasive health inequalities, public health professionals, researchers and non-academic partners in the United Kingdom are mobilising to understand how and in what ways community assets can address health disparities at scale in complex systems. While there is recognition that cultural, natural and community resources can improve health outcomes, these are unequally dispersed with lack of integration in communities and health and social care systems. Researching Evidence-based Alternatives in Living, Imaginative, Traumatised, Integrated, Embodied Systems (REALITIES) is a participatory action research Scottish consortium of 57 with established community asset hubs in five localities with strong relationships uniting conflicting ways of seeing the world. Our collective of lived and felt experience community members, community-embedded researchers, academics and non-academics draws upon a variety of practices, methods, datasets and philosophies to expand existing approaches to tackling health inequalities.

**Methods:**

We present conceptual and theoretical underpinnings for our co-produced systems-level model and empirical findings from testing REALITIES across three disadvantaged localities (November 2022, ongoing). After explaining the context that led to the development of the new scalable REALITIES model for integrated public systems to interface with ‘assets’, we detail philosophical pillars and guiding principles for our model and how we applied these mechanisms to explain how integrated partnership working can lead to improved health outcomes across multiple public systems.

**Results:**

We present a meta-analysis from co-producing and testing the model, showing how measuring change in complex public systems involves critical investigation of People, Process, Place, Price, Power and Purpose. Our critique reflects on power imbalances and inequities in Research-practice-Policy (RPP) partnerships and suggestions for how to nurture healthy ecosystems: overcoming barriers and enabling participation; reflecting on challenges of scaling up, testability and complexity of RPP partnerships; moving from siloed learning to transdisciplinary collaboration in practice; ensuring knowledge exchange has direct impact on communities and frontline practitioners; embedding relational ethics and safeguarding into daily practice.

**Discussion:**

We propose the REALITIES model to unite alternative, sometimes conflicting, ways of thinking about public systems and community assets by continuously reflecting on entanglements between different assumptions about knowledge, reality, evidence, and unnecessary binaries between creative methodologies and scientific method.

## 1 Introduction

Researching Evidence-based Alternatives in Living, Imaginative, Traumatised, Integrated, Embodied Systems (REALITIES) in Health Disparities responds to calls for United Kingdom research-practice-policy (RPP) partnerships to explore different collaborative models for integrating co-production into the improvement of complex systems. Co-production, in this case, refers to equal and active involvement of partners including communities, health and social care practitioners and the voluntary sector in designing and delivering services, programmes or initiatives (1, 2).

Researching Evidence-based Alternatives in Living, Imaginative, Traumatised, Integrated, Embodied Systems is a collective of lived-and-felt-experience community researchers already embedded within neighbourhoods, local council representatives, third sector organisations, Scottish performing organisations, and a Scottish Government executive non-departmental public body and made up of artists, environmentalists, and academics from diverse disciplines including health policy, health economics, mental health nursing, counselling, psychotherapy and applied social sciences, new public management, human geography, environmental sociology, design innovation and participatory design. Our consortium notes a paucity of empirical research identifying systems-level and organisational factors that effect the scaling up of evidence-based interventions (3–5)—fewer still that are community-led and informed by participatory action research (PAR) privileging the person, while recognising systemic and structural inequalities.

Our transdisciplinary meta-model aligns academic, community and public sector values by drawing upon a variety of practices, methodologies and paradigms (quantitative, qualitative, post-qualitative and arts-informed), datasets and philosophies to achieve population health impact across public systems. REALITIES expands existing approaches to tackling health inequalities by offering a fundamentally different perspective on the experience, expression and measurement of wellbeing disparities linked to interconnections between the quality of our relationships, creativity and nature in community settings. It did this in phase 2 of foundational research from November 2022 to January 2023 by bringing together a multi-site transdisciplinary team comprised of 27 people working across three localities in Scotland–Clackmannanshire (Clacks); Easter Ross (ER) in the Highlands; and North Lanarkshire (NL). Through partnership working, we connected with multiple hyperlocal communities and hundreds of community members with first-hand experience of trauma, homelessness, poverty, unemployment, displacement, poor mental health or imprisonment. In phase 3 (February 2023–2027), we are further testing our model at scale in two additional localities namely Dundee and Edinburgh. This brings the total number of our whole consortium to 57.

In this paper, we present conceptual and theoretical underpinnings for our co-produced model and initial empirical findings from testing REALITIES across Clacks, ER and NL. Firstly, we explain the context that led to the development of the new scalable REALITIES model for integrated public systems to interface with ‘assets’ defined as cultural, natural and community resources known to improve health outcomes though usually unevenly distributed over space and time. Such assets include artists and arts organisations; libraries; museums; heritage sites; green and blue spaces such as parks, coastlines and waterways; gyms and exercise-related assets; legal or debt advice services and, importantly, the relationships and research-practice-policy partnerships connecting them.

Secondly, we detail philosophical pillars and guiding principles for our model and how we applied these mechanisms to explain how integrated partnership working can lead to improved health outcomes across multiple public systems. Finally, we present a meta-analysis from co-producing and testing the model in our 3 Scottish ‘asset hubs’ in phase 2 showing how measuring change in complex public systems involves critical investigation of People, Process, Place, Price, Power and Purpose. Our findings show that intergenerational trauma, displacement, social injustice, and the economy are key issues perpetuating health inequalities and poor health outcomes. Our critique reflects on power imbalances and inequities in RPP partnerships and suggestions for how to nurture healthy ecosystems by overcoming barriers and enabling participation; reflecting on the challenges of scaling up, testability and complexity of RPP partnerships; moving from siloed learning to transdisciplinary collaboration in practice; ensuring knowledge exchange has direct impact on communities and frontline practitioners; and embedding relational ethics and safeguarding into daily practices.

We propose the REALITIES model to unite alternative, sometimes conflicting, ways of thinking about public systems by continuously reflecting on entanglements between different assumptions about knowledge, reality, evidence and unnecessary binaries between creative methodologies and scientific method. Our theoretical and empirical contribution reimagines RPP partnerships within and across public systems as ‘assemblages’ or ‘non-hierarchical relational territories and encounters of various human, more-than-human and material entities that influence, affect and are affected by each other’. For these entities—or effective eco-systems to thrive—‘learning takes place between all different parts within the assemblages and is perceived as a non-linear and rhizomatic process’ (6).

## 2 Methods

### 2.1 Context

The Project Lead and several REALITIES consortium hub members have been involved in health and social care integration since mandated by the Public Bodies Joint Working (Scotland) Act 2014—directly through statutory or third sector strategic partnerships informing locality planning processes; indirectly as researchers or practitioners enabling co-designed integrated care services and systems or measuring asset-based, co-produced community-led approaches; or as community members affected by legislation. While supportive of this shared vision for healthier communities and reducing inequalities through person-centred, asset-based and joint-up working, a decade later our team is still navigating complex, fragmented systems within this policy landscape. Feedback from multi-sectoral partners largely remains the same—limited time and resources (if anything, an increasing expectation to do more, faster, with less), speaking different organisational languages with varied, conflictual working cultures; divergent leadership models with wide-ranging views on evaluation; bureaucracy; siloed thinking; asset-based approaches dubbed an exploitative, neoliberal tool with communities and grassroots organisations expected to do more without sufficient resourcing; narrow conceptualisations of evidence (statistics count more than stories); and epistemological and methodological incompatibilities (research needs to produce a particular kind of knowledge that is ‘scientifically sound’ and ‘valid enough’) (2, 7–10).

Community members, particularly the most vulnerable, still feel engagements and consultations are largely tokenistic and piecemeal; over-researched with academics, statutory and non-statutory bodies ‘parachuting in’ to complete reports and tick boxes that do not result in meaningful change; under-supported and abandoned (2, 11). Austerity measures have exacerbated disparities disproportionally affecting the unemployed, isolated and ageing populations (12, 13), and professionals working in this environment to address widening health inequities are reporting burnout, vicarious trauma and compassion fatigue (14). COVID-19 has further fragmented our fragile wellbeing ecosystem. From an ecological viewpoint, public health’s four dimensions are in misalignment. We need to bring ‘material’ (life-sustaining physical building blocks); ‘biological’ (bio-physiological processes including animals and plants); ‘cultural’ (interpersonal relationships, community, family and rituals); and ‘social’ (institutions and legal frameworks) into balance. Within this context, how can we work differently, imaginatively and collaboratively to integrate co-design and co-production into the improvement of health and care complex adaptive systems? We propose The REALITIES model.

### 2.2 The REALITIES model

Our starting point is accepting we are part of a fragmented, traumatised system. We are guided by Karen Treisman’s seminal work on organisational trauma conceptualising the system as the ‘client’, ‘service user’, ‘vulnerable participant’, ‘patient’ or ‘deprived person’ with ‘lived experience’. Burnt out and suffering from compassion fatigue, the traumatised system polarises *people, places* and *processes*. It is crisis driven; avoidant or detached emotionally to cope with insurmountable global inequities. It is chaotic; dysregulated; disconnected (15).

Our team’s work and existence in ‘fragile’ communities also evokes a sense of the system being grief-stricken, lost and alone. Through a place-based lens, we are witnessing neighbourhoods in mourning through, for example, de-industrialisation, derelict sites and scars within rural areas from highland clearances leading to loss of identity and disconnection from the land and its natural assets. These places and local systems may require rehabilitation or healing. This could involve reconciling the past through cultural memories as a form of resistance or transformation. Rebalancing access to natural assets and integrating them into asset hubs also requires assessment of our own positionality within nature-human *relationships*—the extent to which natural assets or resources are considered as commodities compared to their inherent value in nature beyond metrics attributed to them within existing structures and systems. Furthermore, investigations of *power* equity and equitable resource distribution—how natural assets could be organised in healthy integrated systems, for example, through self-organisation (16) and consciousness-raising processes that supports people to challenge existing local governance arrangements and increase meaningful involvement in decisions over natural resources (17).

To bring about systemic change through conscious and co-ordinated engagement in hyper-local communities, REALITIES connects internal systems (individuals’ inner worlds, subconscious motivations, subjective experiences of reality) to external systems (complex, ecological public health) through a multi-faceted approach that connects *people, places, processes* and *power*. An integrated, healthy system needs to understand and reconcile divergent views of reality (ontology); knowledge (epistemology); and vulnerability (relational ethics) through methodological convergence (or find ways to integrate methodological divergence) that situates participatory, arts-informed, creative-relational, (post)-qualitative approaches alongside scientific, biomedical, positivist approaches in the evidence-base. This requires *deep, slow, immersive, critical thinking* with community members (especially the so-called vulnerable) alongside practitioners embedded in communities as well as decision makers enacting policy. It also calls for academics across the disciplinary spectrum to come together with open minds to acknowledge different types of knowing (18), as knowledge and evidence means different things to different people.

Any consortium seeking to achieve effective change must take into account multi-layered, multidimensional factors linked to these foundational values and cultures within and across organisations and communities (19) and acknowledge the complexity of exposing each other to ‘alternative ways of seeing and interpreting the world’ in integrated systems that amass professionals, practitioners, services and community members from established silos (20, 21). To this end, creative-relational inquiry has been proposed as a conceptual frame for whole-system integration. Embracing both population-based and person-centred approaches to public health, it encompasses multiple needs of whole populations rather than so-called deprived communities, care groups or diseases, and places the creative and relational at the heart of integration. While health policy and practice has been largely grounded in reductionist principles that privilege scientific evidence over lived experiences (for example, use of statistical evidence and formulae to determine central funding to local authorities) (22), there is now a shift towards acknowledging and integrating our different ways of seeing, being, knowing and doing (9, 23).

Through this conceptualisation, leaders and frontline staff working in integrated settings with communities value creativity and their own (and others’) ‘situated, positioned, context-sensitive, personal, experience-near, and embodied experiences’. They also connect these experiences to ‘the political, the social, and the ethical’ thereby problematising ‘agency, autonomy, and representation’. Furthermore, we have a shared understanding of integration as a relational process that ‘is dialogical and collaborative’ and ‘explicit and curious about the inquiry process itself’. Our RPP partnerships connected to community members’ lived and felt experiences are the lifeblood of our consortium providing close-up explorations of complex relationships in multi-disciplinary asset hubs through participatory approaches, the arts and performance as methodology—alongside statistical datasets (2, 24).

To address health disparities, REALITIES proposes science must go beyond replicating experiments to lock down a novel facet of reality, to accepting that the human experience offers a prism of realities that can be creatively and empirically navigated to produce new meanings in our understanding of health disparities (25). REALITIES has created a practical; intellectual; human, non-human and more-than-human ‘space’ to bring quantitative methodologists and economists into dialogue with community-led humanities and arts-informed methods and theories in the social sciences. We have also connected the relationships and knowledge developed through the REALITIES model with alternative ways of managing health and social care activities in practice and policy ‘spaces’ moving away from the current dominant New Public Management paradigm characterised by 3Ms—Markets, Managers and Metrics (26)—which hinders the implementation of relational and person-centred healthcare to a Human Learning Systems (HLS) approach to public management (27).

### 2.3 Our three localities and their ‘outliers’

Our team developed and tested the new scalable REALITIES model integrating systems to interface with community assets by connecting to ongoing work in three Scottish localities (growing to five from February 2023 when Phase 3 began). These became ‘asset hubs’ as fieldwork progressed.

North Lanarkshire is Scotland’s fourth-largest local authority with a resident population of around 340,000; the highest rate of school exclusion for looked after children; and 24.8% of children living in poverty (the national average is 23%). 21,500 of NL’s residents live in the 5% most deprived areas; and 75,000 residents live in the poorest 15% data zones (The Plan for NL, 2021).

Clackmannanshire, with a population of around 51,000, is known as the ‘wee’ (sometimes forgotten) county; the smallest historic county in mainland Scotland; despite diverse agricultural initiatives built on reclaimed land and rich alluvial soil, agriculture suffers from land subsidence caused by coal mining. 27% of children in Clacks live in poverty. It is described by locals as badly neglected with poor transport links; blighted by poverty yet abundant in creativity; surrounded by absolute beauty, tranquillity and potential.

Our third locality in REALITIES phase 2 is Easter Ross with around 9,000 residents and a large disparity between villages and towns; loosely defined area in the East Ross-Shire area of the Highlands. ER encompasses towns like Alness, Tain and Invergordon. All three have large housing estates; both Tain and Alness have regeneration ‘projects’; Alness and Invergordon have major issues with National Health Service (NHS) dispersion services with doctors surgeries not seeing patients for 6 months due to staffing shortages; locals struggling to get prescriptions; severe health crisis; high unemployment; low educational achievements; high disability rates; transport problems due to poor infrastructure, high costs impacting on employment and limited timetables since COVID-19 restrictions. ER is behind the Scottish mainland for innovation and growth with finances dwindling since Brexit. During the Syrian evacuations, the Scottish government housed refugees in the small town Milnafua, adding to the estate’s complexity.

Our fourth asset hub in phase 2 was called ‘The Outliers’ with a focus of integrating excluded populations, in particular ex-offenders, into our localities and systems. A person or group detached from the system, outliers have significant impact on statistical analyses and skew results of any hypothesis. These excluded individuals or communities offer ‘extreme values’ that differ from most other data points. They require careful consideration when generalising and trying to make sense of root causes of health inequalities due to a combination of complex, systemic and unique, personal circumstances linked to exclusion. In REALITIES, we do not view ‘The Outliers’ as separate from the system but integral to it. We worked with a charity and community-embedded research with access to prisons and other displaced or excluded populations to explore how or if prisoners, ex-offenders, refugees and those experiencing homelessness are integrated within statutory and non-statutory services and RPP partnerships.

### 2.4 Means of data collection

Each asset hub was comprised of a non-academic community-embedded researcher from the third sector or local authority connecting to a network of lived experience practitioners already embedded in each locality. ‘The Outlier’ community-embedded researcher provided insights cutting across all three geographic sites. They all worked alongside four Early Career Researchers (ECRs) from diverse disciplines mentored by senior academics. ECRs were supported to take the lead on co-producing the structure of a series of participatory action activities, events or workshops to be systematically captured across the sites using the REALITIES model.

While the content and format of these engagements were different depending on the needs, wants and circumstances of local communities, each session held these primary objectives in mind. The REALITIES Consortium sought to:

- map a detailed understanding of the range of services, scale of provision, key stakeholders and existing partnerships (statutory and non-statutory), using bespoke participatory methods and tools, to establish clearly articulated asset hubs;
- expand existing partnerships through new collaborations cutting across academia, health and community partners, funders, lived experience practitioners and policymakers;
- engage with lived experience practitioners in whatever manner was fitting for that community to help test the REALITIES model (this could range from co-creating theatre in prisons to understand re-integration into communities after liberation; to creative, participatory sessions in nature to explore how community members’ feel about their sense of place and how this in turn affects their wellbeing);
- connect cultural, natural, social and creative-relational assets rather than viewing these as separate entities; and
- expose our RPP partnerships to different methodological approaches and disciplines, and develop new capabilities and understanding of knowledge creation, data and evidence.

The REALITIES consortium commenced data collection through a 2-day, immersive residential in February 2023 where we shared our diverse backgrounds, disciplines and asset hub contexts; delivered participatory action sessions on co-design; HLS; relational ethics; dance and other embodied practices; arts-based approaches including community theatre; immersive nature-informed creative writing as we walked through a rural setting; and place-based methodological tools. This foundational gathering set the scene and tone for a collegial partnership network that valued relationship, understanding, shared purpose and commitment to working together and promoting healthy relational dynamics despite divergent views and approaches.

We then embarked on a series of 45 emergent, creative participatory action workshops (often in nature) co-led by marginalised groups with partners and community-embedded co-investigators gathering and co-synthesising data from hundreds of people in deprived communities (see Table 1 for a summary). We collected data in a range of ways including interviews; reflective diaries (ethnographic notes); photographs; and art (visual, performative and audio). Core team members also met regularly for ‘check-ins’ and debriefs to facilitate sense-making and meaning-making sharing what was *not* working, and reflecting on the barriers and challenges of the task at hand. ECRs also met with the Project Lead and four senior academics for four 3–6 h PAR and training sessions on integrating health economics; quantitative datasets; HLS and co-design into our community-led fieldwork.

**Table 1:** Data collection for REALITIES phase 2.

Project Stream	Who?	Why?	What?	How?	When?
	*Definition of Community*	*Reason for intervention/evaluation, and why implemented at this time?*	*Description of intervention/evaluation*	*Means of data collection*	*Timing of intervention/evaluation*
Recovery Cafes in NL	Various recovery groups across North Lanarkshire (NL) (80)	To support those in recovery from addiction by offering creative support.	Co-produced creative zines with those in recovery from addiction.	Creative material and community embedded researcher ethnographic insights from the field.	February 2023–July 2023
REALITIES session with OYCI in clacks	Young people who attend the outdoor ‘Stress Free Sunday’ sessions with OYCI in Clackmannanshire (6)	Exploring the synergistic benefits of combining creative-green activities.	Creating illustrations using watercolour pencils in an outdoor session in a woodland area.	Arts-based participatory action-research workshop.	March 2023
		Allowing young people to be creative and make friends in an environment that may not be readily available them.	Wider programme: Relaxed outdoor sessions learning about the natural environment and bushcraft.		
REALITIES session with WEA in Easter Ross	Mental health group in Alness, Easter Ross (6)	Exploring the synergistic benefits of combining creative-green activities.	Painting stones to make markers for the plants the group had been growing in the garden.	Arts-based participatory action-research workshop.	June 2023
		To build confidence and motivation to be around other people; develop skills and attitudes needed to seek and gain employment.	Wider programme: Growing/gardening project at a formerly disused greenspace.		
REALITIES networking event	Partners in Highland (33)	Event that drew together all partners in REALITIES in Highland.	Event in Invergordon that drew together all our partners in Highland. It was an opportunity for everyone to meet and discuss their input and future ideas for next phase.	Networking, notetaking	July 2023
REALITIES session with Scottish Opera, WEA and Support in Mind in Alness	Mental Health group in Alness, Easter Ross (10)	Exploring the benefits of music and creative arts for mental health recovery.	Explored opera in an outdoor setting. Painted a group picture to coincide with the music. One-off programme to introduce opera to new listeners.	Arts-based participatory action-research workshop.	May 2023
Creative Families REALITIES sessions	Community family arts project in Invergordon (23)	Exploring the arts in family groups.	Different art mediums were employed to bring families together to do art activities in a local community centre. Participants got to choose the medium every week.	Arts-based participatory action-research workshop	June 2023
Working with Others	Mental health group in Alness (8)	Group award to build confidence in working in a team.	The group built a sensory garden for the local community. They worked in an outdoor reclaimed space and grew vegetables in a formally disused greenspace.	Arts-based participatory action-research workshop.	June 2023
Positive Moves launch in Clacks	Practitioner, third sector, research and community members (80)	Session on Clackmannanshire’s new programme to support local people seeking work or who want to take their first steps back into employment.	Reflecting on how positive moves fits in to the existing Clackmannanshire Employability System and support it offers individuals aged 16–67 in Clacks.	Focus groups, informal discussions, networking, and reflective notes.	April 2023
OYCI summer prog-ramme	Young people in Clacks aged 10–14 years (15)	Delivery of green spaces session for local young people.		Creative sessions in nature.	July 2023
Release Reimagined (The Outliers)	Bethany Tenants	To support those in supported accommodation and with experience of homelessness and/or custody sentencing and/or addiction, by offering creative outlets.	Creative story-making process, which invites participants to reflect their experiences and hopes through the development of a collective fictional narrative.	Workshop diaries by community embedded researcher	May 2023–July 2023
	These are supported tenancies for those who have not held their own tenancy before or for a long time. All are prison experienced (7)	To explore the meaning of release in the context or marginalised communities and those ‘hard to reach’ through place-based interventions.		Photographs of the notes and drawings made in each of the workshops	
				Final story	
Release Reimagined (Outliers)	Inverclyde Recovery Hub – Your Voice	To support those in supported accommodation and with experience of homelessness and/or custody sentencing and/or addiction, by offering creative outlets.	Creative story-making process, which invites participants to reflect their experiences and hopes through the development of a collective fictional narrative.	Workshop diaries by community embedded researcher.	June 2023–October 2023
	Your Voice is a lived experience network for people who have recovered or are recovering from a place of dependency on alcohol and drugs, based in Greenock. Eleven people (including two volunteers) have taken part in this group (11).	To explore the meaning of release in the context or marginalised communities and those ‘hard to reach’ through place-based interventions.		Photographs of the notes and drawings made in each of the workshops.	
				Final story	

These sessions took place alongside desk-based analysis of issues for a social statistics approach to area health effects research. We explored official statistics on under-represented groups, and investigated how definitions of ‘value’ and ‘quality of life’ in traditional health economic models are not necessarily aligned with experiences of our most deprived communities. We identified that social aspects of place are poorly captured in official neighbourhood statistics yet are known to have an independent association with health outcomes (28).

### 2.5 Participatory action research meta-analysis through human learning systems

We conducted PAR within and across our multiple asset hubs applying HLS as an overarching methodology which embraces the complexity of the real world, and enables us to work effectively in that complexity (27).

Participatory and collaborative by nature, PAR calls for equal and active involvement from communities of research who gather data with academic researchers and are dynamically involved in co-analysis—or sense-making and meaning-making—through a cyclical process of action, reflection and collective inquiry repeated until patterns emerge (29). The approach flattens traditional epistemological and ontological hierarchies in the process of evidence creation, as different versions of knowledge and reality emerge and are given equal weight until resolutions to research problems emerge. This process simultaneously addresses power imbalances in academic discourses and datasets by rendering those with lived and felt experiences of a particular issue as experts, rather than academics.

Through deep listening, reflexivity and conscious reflection on shared processes together, the REALITIES consortium reviewed how our diverse positionality, context, behaviours, disciplines and paradigms shaped the emergent construction of knowledge. Embracing messiness, complexity and relationality, we continuously and consciously interacted with the academic literature, existing datasets, theories, conceptualisations and our own experiences to generate knowledge and new meanings (30).

This approach, which privileges long-term, mutually beneficial relationships between researchers, public sectors and community members, inherently promotes an alternative public management paradigm namely Human Learning Systems (27). As ‘a giant action research process’ with ‘interconnected learning cycles’, HLS is a ‘heuristic device’ supporting systems ‘to move away from a prescriptive programme’ by ‘encouraging learning and experimentation’ (31).

The REALITIES Consortium applied this thinking to the reimagining of health systems, accepting that multiple types of knowledge co-exist and need to be converged to produce new meanings. People’s lived experiences of connecting to wider social, economic, political, geographical, affective and creative contexts is central to changing practices and impacts in communities. Through our co-analysis, we sought to develop new ways of working across disciplinary and organisational boundaries to provide public (or alternative) services improving the wellbeing of so-called deprived communities.

From a perspective of complexity within public services, one aspect of a system is connected to other systems on both micro and macro levels, so changes in one part of any system will impact the functioning of other aspects. This helped us understand and unravel links between economic and social inequalities, and uneven distribution of health outcomes within United Kingdom communities, with a view to evenly resource and distribute community assets to improve health outcomes in future phases of research.

Community-level organisations were conceptualised as one level of learning cycles, where each area has developed an understanding of the health disparities experienced by people living there, the current community-based assets available for supporting people to improve on their health, and gaps in the provision. This meant PAR sessions could be delivered as an organic, emergent response to challenges identified by local communities, creating grassroots evidence for developing systems that support local wellbeing, and can be tested in other areas.

We then embarked on a pattern-spotting and meaning-making across all sites, enabling a deeper level of understanding and opportunities to develop further REALITIES sessions linked to emergent findings based around community-led needs. This led to the creation of a novel converged database connected to practitioners, policymakers and strategic partners. In this way, we co-produced evidence from the bottom-up and fed it back to local communities, practitioners and policymakers so that new learning cycles could emerge and make a difference to deprived communities in ‘real time’.

## 3 Results

### 3.1 Reframing health and social care systems

**Figure 1:**
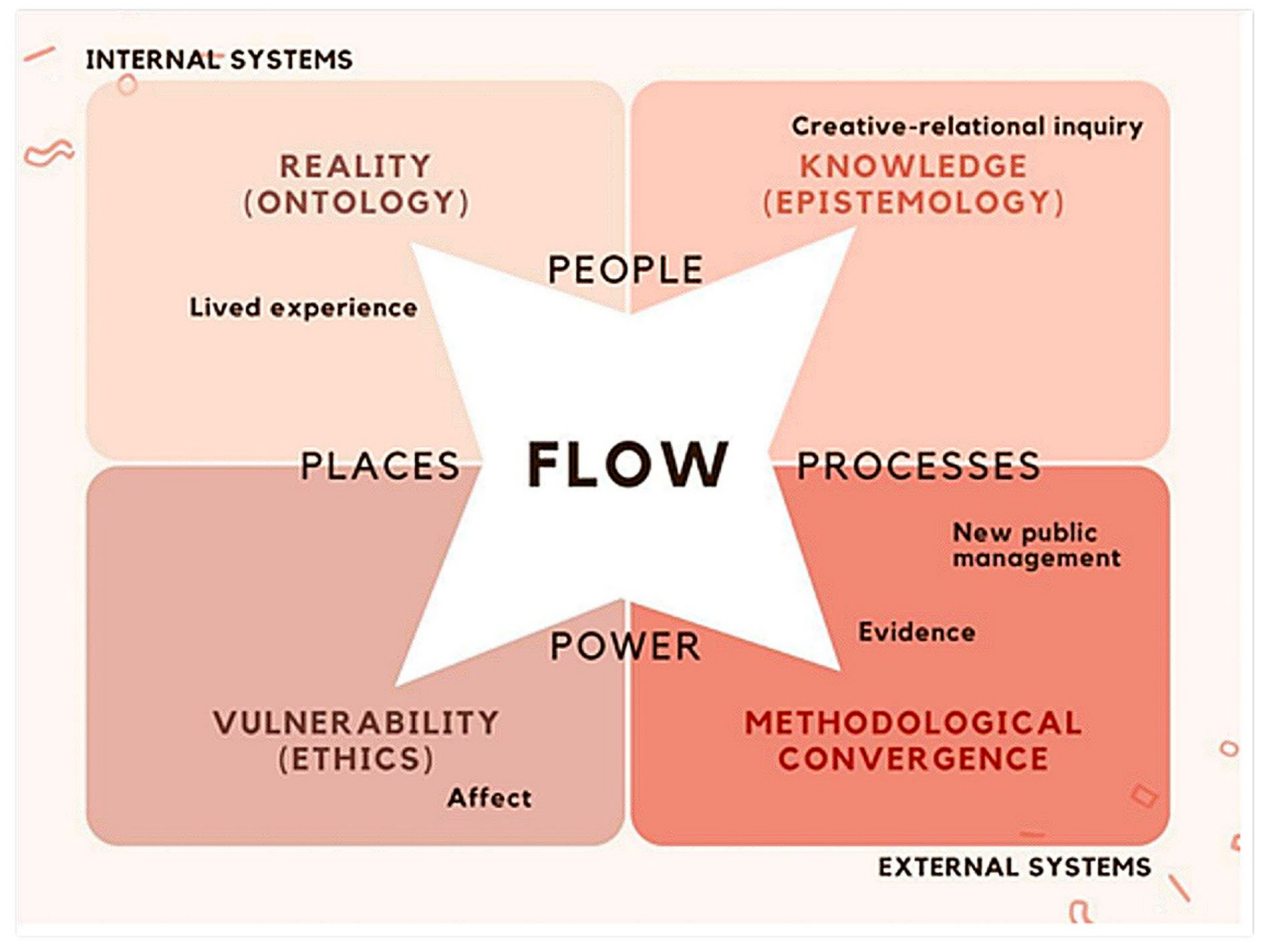
The REALITIES Model – 1st conceptualisation with 4Ps.

**Figure 2:**
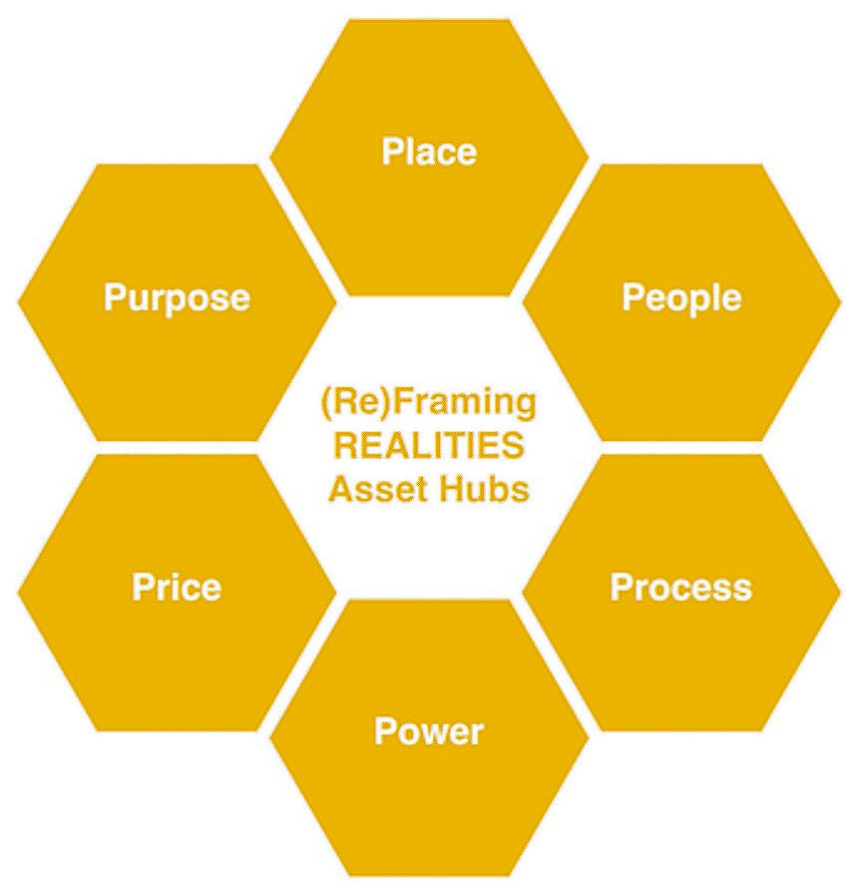
The REALITIES Model – 2nd conceptualisation with 6Ps.

Researching Evidence-based Alternatives in Living, Imaginative, Traumatised, Integrated, Embodied Systems takes a Human Learning Systems approach noting health and social care systems are constructed mental representations of relationships existing in the world to promote health for people. Our multi-faceted model connected People, Places, Processes and Power to think differently about how ‘health systems’ connect with community assets. Through phase 2 fieldwork, 4Ps in our original conceptualisation of the model (Figure 1) became 6 (Figure 2), as Price and Purpose emerged as further guiding principles. Drawing on Patton’s Principles-Focused Evaluation (32), we measured change against how well we adhered to these RPP principles rather than whether or how we delivered or replicated the same activities within asset hubs. This helped us focus on achieving our objectives, while maintaining operational flexibility across our asset hubs.

Our model purposely pushes against the challenge of identifying a universally accepted ‘rigorous’ method of evaluating change in complex systems drawing on extensive research showing there is no one-size fits all—we need to broaden ‘what data we rely on, and how we collect, analyse and present it’; balance our ‘long and short view on change’; and ‘focus on momentum and learning as indications of progress’ rather than linear, traditional understandings of evaluation (2, 33).

**Figure 3:**
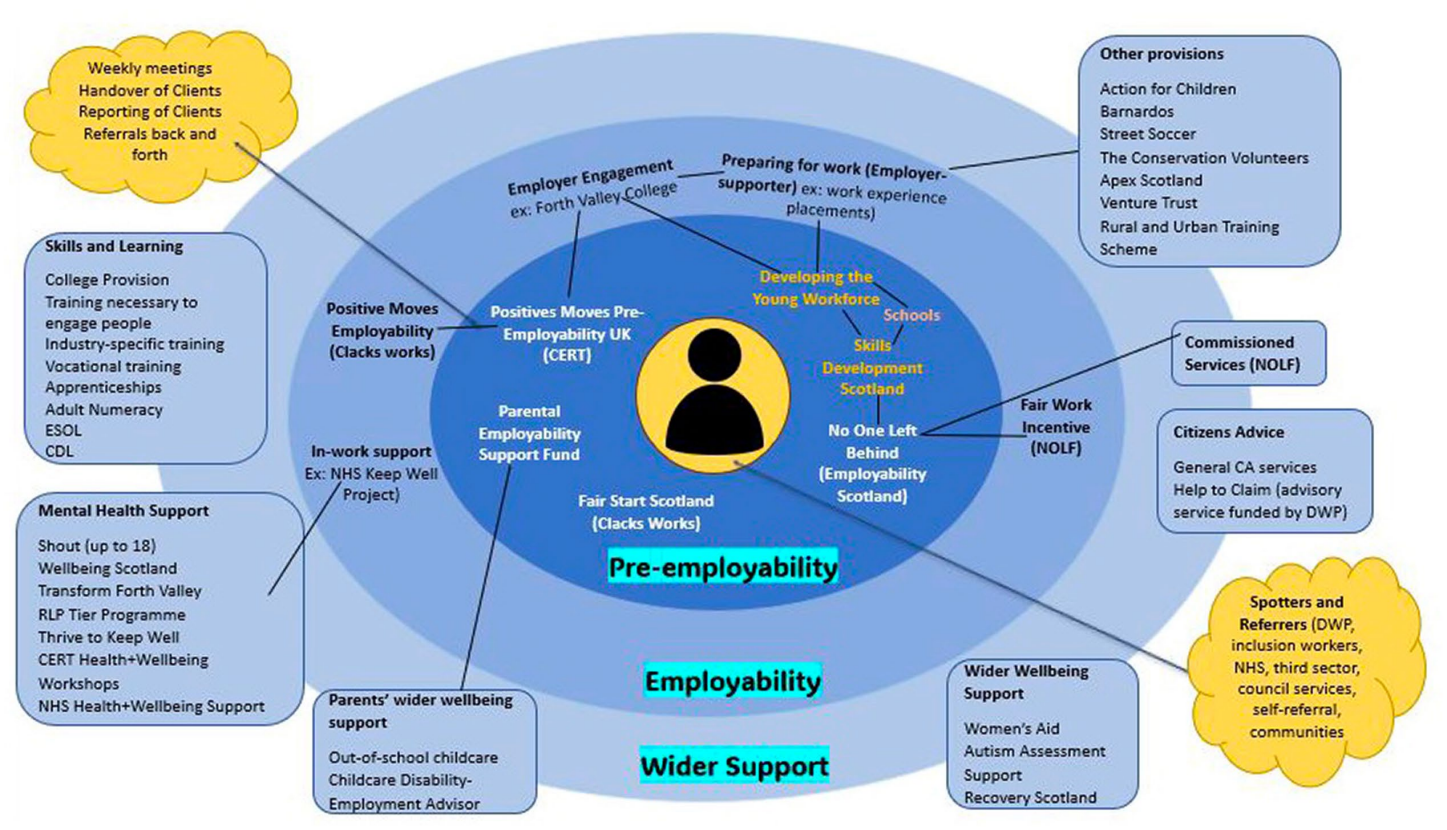
Clackmannanshire asset hub mapping.

The Clackmannanshire asset hub focused on the intersection of health and social issues, primarily employability and youth populations. Employability and *‘pre-employability’* were key concerns for our main community-embedded researcher and local community groups recognising that low employability can be directly linked to health inequalities and lack of access to services (for example, transport). Using a person-centred approach both with service users and service providers, work in this phase mainly consisted of relationship building and networking across service providers in Clackmannanshire to improve delivery. This led to gaining *‘on the ground knowledge’* and mapping community assets (Figure 3).

**Figure 4:**
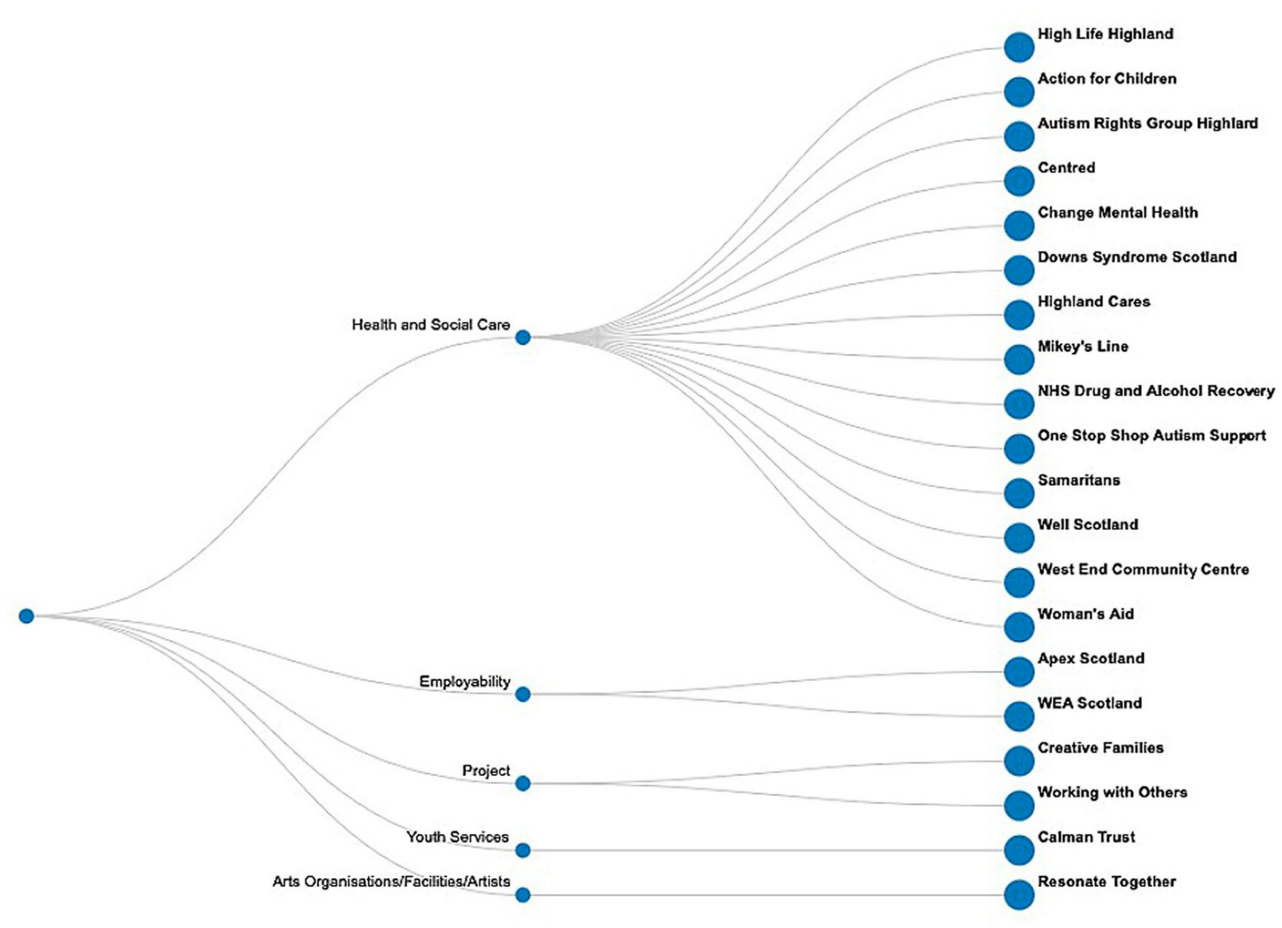
Easter Ross asset hub mapping.

Our ER asset hub focused on community integration and partnerships bringing together a number of public and third sector organisations dealing with a diverse range of social and health inequalities. These include mental health issues, domestic abuse, unreliable public transport, digital poverty, drugs and alcohol use, housing issues, adult literacy, and access to green space. The hub is forging and cementing new and old links across ER to map existing services. In the process, ER has established a ‘*flexible, intuitive*’ hub ‘*accommodating to local needs*’. Mobilising these organisations has created opportunity for research into links between creative and green assets and mutual benefits for community health in a future phase of funding (Figure 4).

**Figure 5:**
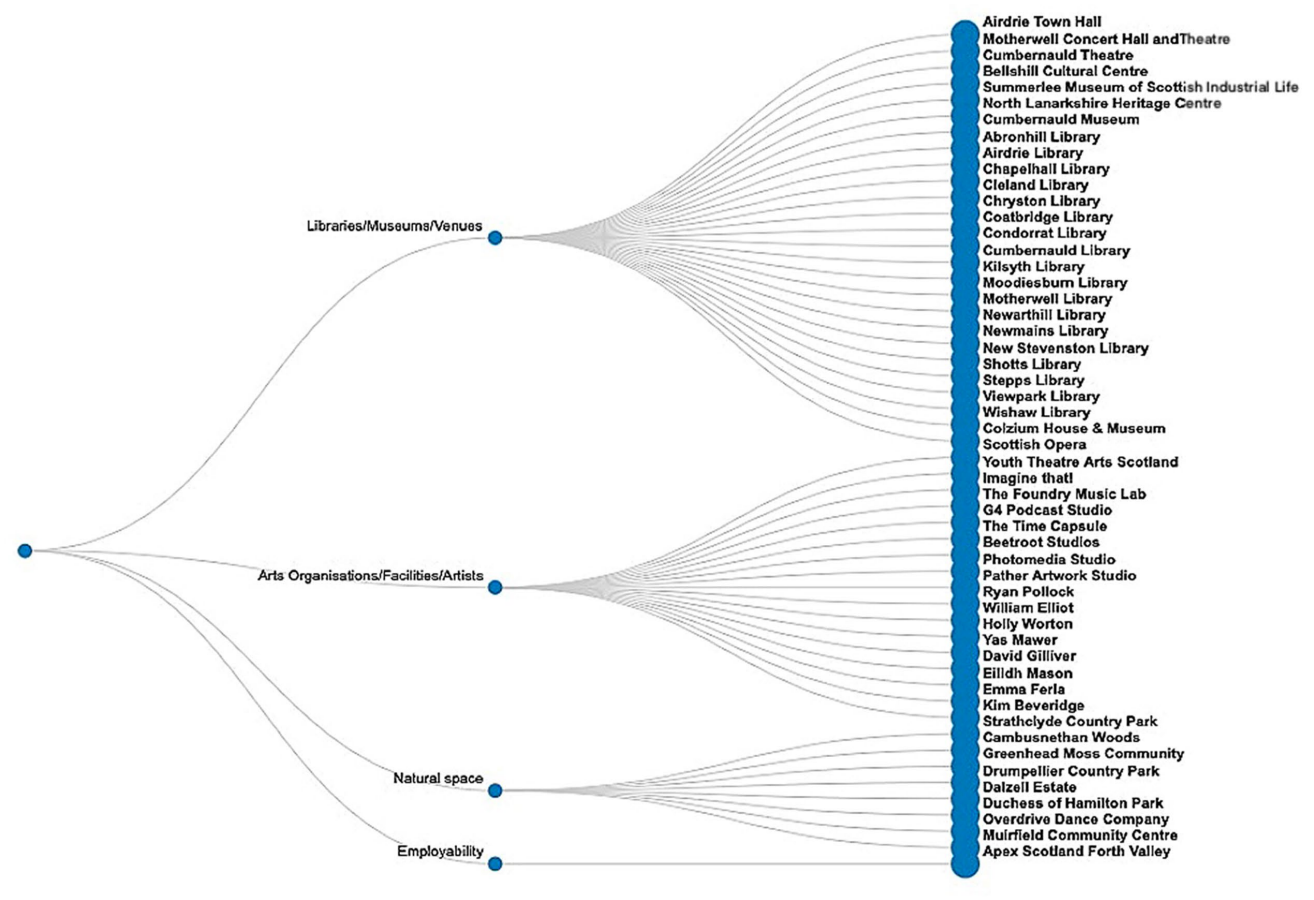
North Lanarkshire asset hub mapping.

The NL asset hub utilised the networks and connections established through a previous place-based United Kingdom Research and Innovation (UKRI) Arts and Humanities Research Council (AHRC) funded project called ‘Art is Everywhere’^1^ to further focus on tackling health inequalities across the lifespan (from early years to healthy ageing) through creative practice and the arts. This place-based project forged strong relationships between the academic team, North Lanarkshire Council (NLC) with multiple other non-academic collaborators cutting across the arts, culture and heritage, social justice, employability, education, health and social care. The intention was to co-produce NL’s first ever strategy to tackle inequalities through the arts with research and practice partners and community members, which has now been published as a five year strategy and is being integrated into council operations across departments and services using a multi-sectoral RPP approach (34). Our RPP also co-produced NL’s first interactive creative asset map made by and for local communities for the council to update with community members as new creative assets emerge. Asset mapping (Figure 5) led to a story-led asset map expressed through local artists and their favourite creative resources in NL [see (35)].

North Lanarkshire’s asset hub supported the formation of an Artist’s Network and our lead community-embedded researcher worked closely with Recovery Cafés across North Lanarkshire to deliver creative activities such as storytelling and zine-making. The goal is to build on this work and widen the scope and impact of the hub to tackle health inequalities in NL by integrating creative practice to engage and work with communities.

**Figure 6:**
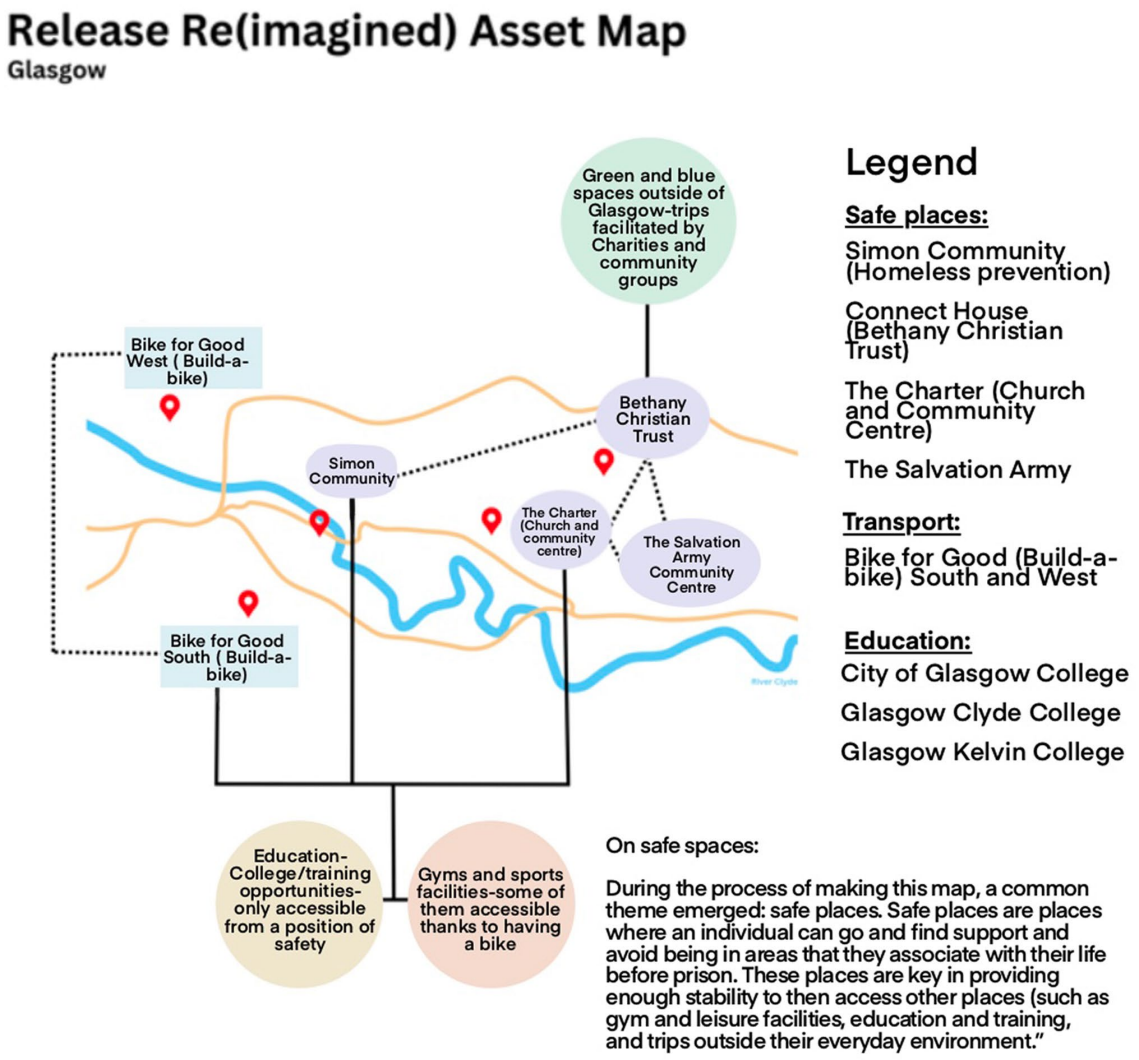
The outlier asset hub mapping.

Finally, our outlier asset hub reached out to people who are often displaced because they have been in prison, have experienced or are experiencing addiction or homelessness, are refugees or seeking asylum (though phase 2 did not specifically focus on communities affected by migration) (see Figure 6). Working with a community partner in prisons and with those who have been released, this hub focused on the theme ‘*Release (Re)imagined*’ seeking to explore what ‘release’ means to each individual.

Our iterative 6P’s model helped us understand how and in what ways community assets are being mobilised in our local hubs to address health disparities.

#### 3.1.1 Place

Clackmannanshire is a rural area interspersed with urban small towns. Many neighbourhoods have hidden poverty and lack amenities and suitable transport links. While rich in natural assets including the Ochill hills and Gartmorn Dam used as sites for our REALITIES creative green workshop, our partners noted that these greenspaces are not used by the local community as there is a perception that ‘these *spaces are not for them but for tourists*’. There are also issues with local transport that can mean ‘*getting to and from the start of a walk can be challenging.*’ The hub has identified further opportunities to connect those seeking work and young people to the natural environment to bring about change. One of the service providers is a youth improvement organisation running initiatives to provide better opportunities for young people. They have their own premises in the town centre for neighbourhoods to easily reach and provide a safe space for young people. REALITIES facilitated sessions on natural assets with young people at various sites in partnership with this organisation, and a future focus on young people and their transitions through engagement with creative and relational activities in nature has emerged as a way of measuring changes in inequalities in phase 3. We will continue to monitor initiatives started during the COVID lockdown supporting the physical and mental health of young people in Clacks addressing issues such as social isolation and ‘*reconnecting with nature and the outside world*’.

**Figure 7:**
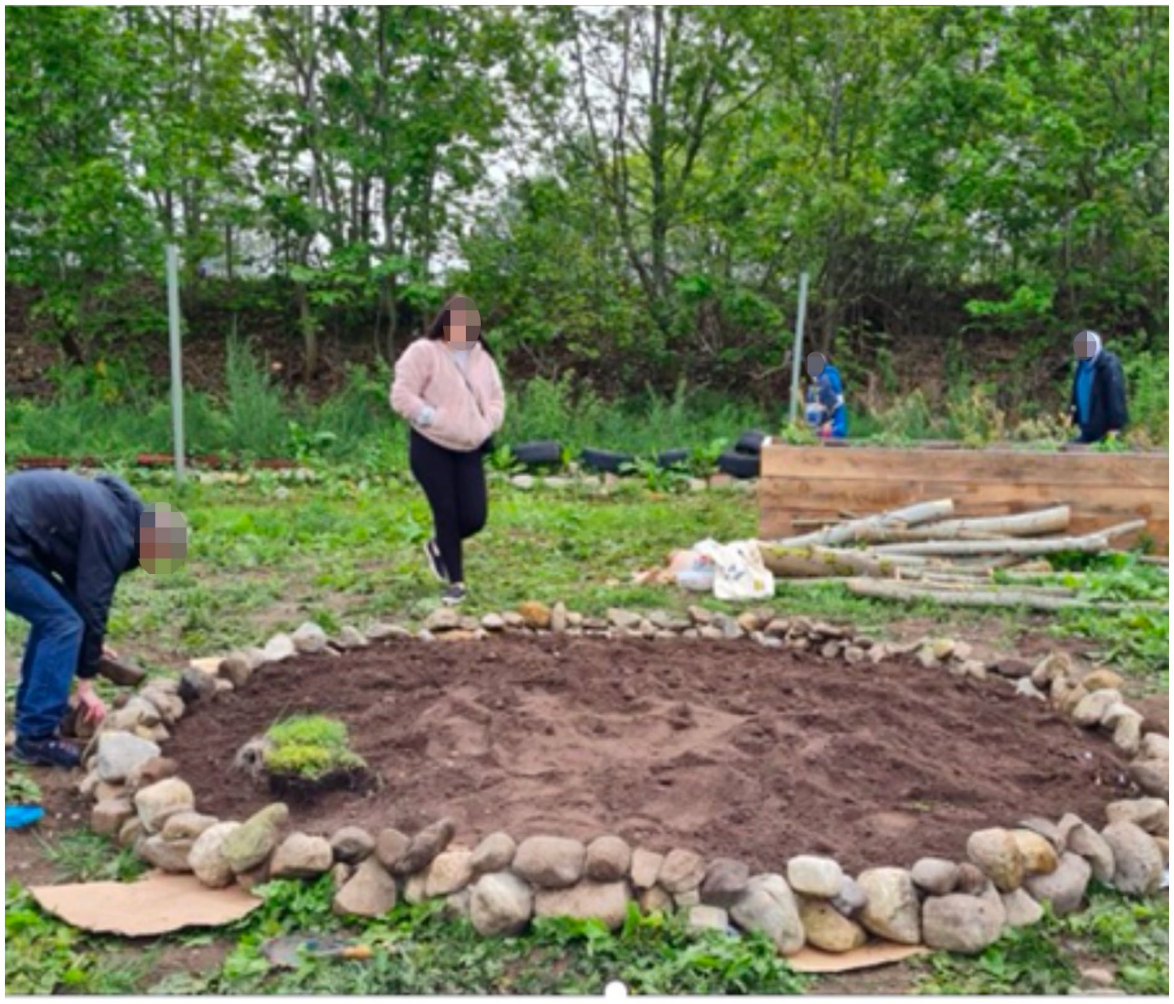
‘The Field’ sensory garden in Easter Ross.

Easter Ross, an area in the south-east of the Highlands comprising a number of small towns, is surrounded by mountains, forests and beaches. Insights from interviews with organisations and reflections from our community-embedded researcher highlighted wide-ranging and complex place-related issues facing communities in ER, including natural assets not being utilised by the local community. There is now an emerging understanding of place ‘being *fluid and given more or less importance based on how it is introduced and maintained*’. As part of this phase, people in ER were provided access to a sensory garden and greenspace known as ‘*The Field*’. They now have ownership of something that they did not have before—‘a *micro connection in a macro environment*’ (Figure 7)

Place is defined as the council boundaries of North Lanarkshire Council for this asset hub with a local Recovery Cafés being key collaborators in this research phase. Once again, a lack of transport infrastructure emerged as a key challenge. For example, a participant described their journey to reach a Recovery Café included ‘*two buses, a long walk, and a train*’. Other participants noted that deeper engagement across multiple cafés was thwarted by weak transport links. Our work in this hub identified opportunities for more integration with other classes delivered by the council, for example, the Artist Network could feed into school workshops to engage practitioners and community members engaged in NHS Health Improvement initiatives thereby weaving tighter links between these three communities and systems.

Finally, our outlier hub challenged the traditional notion of ‘place’ by engaging with displaced communities several of whom have always lived in the local areas, with a smaller number of residents having immigrated into it both nationally and internationally. Exploring the notion of a ‘transient’ asset hub, with people more likely to flow through it due to enforced movement, was initially challenging though in arts-informed sessions it became apparent that place is experienced through a metaphor of ‘*layers*’ and ‘*nodes*’. Participants’ relationship with place often changed after being released from prison. The character from their co-created story, for example, moved through the same area of the city he had been in before going to prison, but did so in a different way—‘*in a different ‘layer’*’—avoiding the places and people that he would frequent and had relationships with before prison thereby re-learning to experience and relate to place in a different way.

#### 3.1.2 People

Through our community-embedded co-investigator’s work in partnership with other local organisations, an asset hub has been established serving and benefiting a wide variety of populations in Clackmannanshire including LGBTQ+, refugees, ethnic minority, men, women, socially disengaged, care and trauma survivors, people who are disabled and ex-offenders. By March 2023, a local charitable consortium had engaged 325 service users with the possibility of hundreds of different services made accessible through them, and database of service providers was designed to link service users to local community assets more easily. Since their inception and until 2022, the consortium has had 1,082 ‘*participant experiences*’, with young people across all demographics typically aged 10–18, creating opportunities for young people in Clacks and supporting creative practice, active decision making and building self-esteem.

Meanwhile, the Easter Ross asset hub served local people by being ‘*a focal point and point of access and integration to a wider web of services*’. Our community-embedded researcher and partners mapped local providers and brought them together to share knowledge and contacts to establish new ways of partnership working. The communities who benefit from engaging with this hub are affected by serious and wide-ranging health issues with mental health highlighted as a particularly key area of concern, underpinned by a lack of meaning and motivation, and a perceived lack of opportunities available. The pillar of ‘People’ in our model was highlighted as being of particular importance, primarily in relation to service providers. It was repeatedly reported that ‘*services functioned well – or badly – depending on the people involved*’. There is a need for the asset hub to approach communities sensitively, as there is an understandable lack of willingness to engage with any initiatives perceived to stem from the same system that many appear to be accountable for the issues community members face in relation to poverty and inequalities.

In NL, Recovery Cafés were delivered by communities with support from the council and creative practitioners to assist in activities. Members were signposted through a number of routes, for example, referrals from Alcoholics Anonymous; Cocaine.

Anonymous; Narcotics Anonymous; word of mouth; and digital platforms (mainly Facebook). Many participants shared stories of ‘*being brought by a friend*’ or ‘*bringing a friend*’ to help them on their recovery journey. More generally, the NL asset hub worked with early years to school children, college students, ageing demographics, people from multiple ethnicities, people in care, alongside neighbourhoods and communities in NL and local place-based artists and Artist’s Network to tackle inequalities through multiple, well-established RPP partnerships.

Creative sessions in our Outlier hub delivered two participant groups in Glasgow and Inverclyde. The former group are residents in supported accommodation intended for ‘*people needing a fresh start and a place to call home*’, for example after leaving prison or having experienced or being at risk of homelessness.

#### 3.1.3 Power

Team members (despite specific disciplines and varied backgrounds) are trans/multi/interdisciplinary with experience of working in mixed teams and beyond their professional boundaries and comfort zones daily. The management action in place for execution of the project combines the team members’ expertise to complement each other’s work. Our diverse expertise means we do not need to ‘outsource project activities’. Our approach also allows for a strong degree of autonomy, collaboration and respect that transcends the classic Project Lead and line manager-based approach typical of most standard research projects.

Our Clacks asset hub reflected on power dynamics at a strategic level highlighting that while service providers strongly feel that they have the right people delivering services, there is a ‘*frustration towards current processes and the lack of power as to who can change processes that are not working for service users and wider communities*’. There has been strained relationship between the council and service providers regarding delivery of services and lack of resources. A lack of power and influence amongst young people has also been highlighted. Our community youth organisation has created opportunity for young people to influence local decision making, for example at the community council, as well as changing perceptions of young people and seeing them as the creative change makers. The initiative uses creative activities and/or the arts to promote community action, campaigning and citizenship through influencing. Projects focus on how much power young people have on influencing issues on topics such as body image, food poverty, cost of living crisis, loneliness and social isolation.

Power—or powerlessness—emerged as a dominant theme for our ER asset hub, particularly in relation to people’s perceived ability—or inability—to change their own lives. Throughout interviews, a key finding was mistrust in central government and unwillingness to engage with ‘the system’. This was also reflected in interviews with third sector organisations, several of whom expressed frustration at being unable to continue work due to cuts in funding and changing governmental priorities. As such power within the asset hub is devolved, with all service providers being introduced and connected to each other. The REALITIES community-embedded researcher and partner organisation continue to act as a focal point to facilitate connections.

In our NL asset hub, risk averseness and lack of willingness to embrace the unknown were highlighted as potential barriers in the council. It was also noted that the policy that community centres can only be opened for council activities when another private hire has been booked through a profit-making company ‘*has robbed the team of access to spaces, making running of activities very hard*’. There is also a perception that bringing people to events in some areas is hard because ‘*people here are very territorial*’. The well-worn consultative pattern of presenting ideas for people to respond to was also highlighted as a challenge, as it avoids sharing of power and misses a genuine exploration of what matters to people and how their energy and ideas could be supported in order to flourish. There is also a need to be mindful of and reflect on emerging negative perceptions that community centres will be closed and replaced with a ‘*one stop shop, super hub*’ that does not meet community members’ needs and wants. Power in Recovery Cafés is enacted through core members influencing newer members and encouraging them to ‘*stay clean, accept mistakes and stay engaged with the group*’. People use their power of local knowledge to connect others to services, and feel empowered through voluntary engagement aided by an informed network of services collectively held by participants and its leadership team.

Power imbalances cutting across and within institutions and fragmented systems—and the structural, historical, social and economic inequalities experienced by those who are most excluded—are reflected in the nature of the outlier hub. Evaluation of creative workshops highlighted power dynamic between the facilitator (a community-embedded practitioner), support staff and participants. While sessions were designed using a participatory approach, themes were determined prior to involvement of groups. Community members note their co-created stories reflected their ideas and discussions, and the process of sharing experiences through storytelling was safe and comfortable. There is opportunity to explore ways to create equitable spaces by further understanding the ‘*tension and balance between support staff offering ideas to develop an encouraging and open space versus speaking on participants’ behalf*’.

When asked ‘*what a system is meant to do and what prevents it from working well?*’, one outlier community member flagged up the ‘*benefits system*’ as it was meant to ‘*help with money, safeguard and recognise differences in people’s situations*’. They noted several systemic issues that stopped it from working well including ‘*not enough money to survive – people turn to other means; no address, no pay – hard when homeless; long set-up time – weeks of living off pittance/building up debt; not set up for the vulnerable*’.

#### 3.1.4 Process

There is a desire within partner agencies in our Clacks hub to streamline processes with bureaucracy noted as a barrier to both process and culture. There were reports of some organisations ‘creating more barriers when solutions were offered’, and a longing to use appreciative approaches to explore solutions and assets that enable agencies to ‘flow through barriers rather than get stuck within them’. A key issue identified was people’s access to agencies and lack of clarity around referral routes. This was attributed to a lack of structure, and the necessity for a single point of contact for service users. Community organisations using outcomes-focused methodologies to track service users’ journeys found these to be generally effective, but there was a wish to explore limitations of this methodology and how it can be improved when converging quantitative and qualitative evidence for evaluation.

Communication between partners and colleagues was highlighted as key to the process in ER asset hub, which has ‘opened lines of communication with groups that have never been connected before’. This hub advocates for bringing people together to encourage change through collaboration and creates opportunities for face-to-face interactions. Our community-embedded researcher noted connections between different organisations was key and effective interventions and change was usually achieved because ‘someone happened to know someone who was willing to do them a favour or meet to speak to them’. Conversely, a breakdown in process often seemed to be underpinned by a lack of connections.

The process of the Recovery Cafés is peer and community-led, with support from NLC and Artist’s Network. The process is ‘organised by people in recovery’ and there are a number of activities that take place in these spaces including guitar lessons, meditation, hot meals, and ‘smart’ meetings (which are a form of check in). Participants work their way towards volunteering and earning qualifications, advancing to training courses to become peers who are trained to work in the service and eventually deliver sobriety workshops in contexts such as prison communities. This journey involves several third sector partners and aims to eventually get participants into paid employment by building up their CVs as part of recovery. It was also found to be an effective way to help keep people attending sessions regularly; build people towards recovery in fellowships; and has enabled more informal and social support that ‘*can feel less intense*’ and are intended as ‘*a stepping stone*’. One community member described it as being ‘*a great opportunity to give back to those who have given so much to them*’. The social and physically active aspects of the sessions prepare participants for the more formal ‘smart’ meeting check in at the end of the day, which supports people to reveal how they are feeling, often seeding their feelings in conversation with others (fairly emotionally to their closest peers). Our NL community-embedded researcher noted ‘*this is a very social, very intimate, very physical, and very active group. There is enormous care between participants*’. In the Artist’s Network, a process is unfolding whereby there is collective agreement over professional and social aims for artists in the local area and how allocated money could be used to impact social inequalities in NL and improve arts provision across the place.

The outlier hub used participatory and creative processes to understand how participants make sense of the theme of ‘release’ and what ‘systems’ can support them in their experience of release. Participants were invited to reflect on experiences and hopes through a creative story making process in the development of a collective fictional narrative. Using storytelling methods during seven weekly two-hour workshops, participants created a fictionalised story about a character that has been released from prison and is trying to find their way outside. One community member commented: ‘*this was a great workshop, and it was almost a shame that it had to end when it did, everyone was clearly enjoying themselves developing the characters and the stories*’.

This process has grown out of our community partner’s creative expressions work in prisons so buy-in or ‘recruitment’ was not an issue as there was a pre-formed group of individuals with established partner relations and interest in participating in creative activities. Reaching out to other organisations where such groups had not yet formed proved unsuccessful, further demonstrating the importance of solid RPP relationships. The final creative output was publication or zine sharing their stories, along with reflections on the process and artwork by those taking part.

#### 3.1.5 Price

There is a shared belief amongst partners in the Clacks hub that many services already exist, but need funding to grow so ‘*the focus needs to be on resourcing the services available rather than creating more services, which may also be underfunded*’. Morale amongst partners was noted to be low as local agencies are being asked by the council to solve what could be considered state or public sector wide problems, without allocating sufficient funding. A lack of investment in transport and recruitment of support workers were also highlighted as key issues. High transport costs have resulted in people being excluded from jobs that are not walking distance leading to lower employability. It also emerged that price or money intersects with the notion of trust. As one practitioner noted: ‘*when there is an increasingly competing demand for resources it can impact trust and result in territorial behaviours*’. Sustainability of projects were also seen as at-risk causing anxiety that funding streams will run out. A co-exploration between partners to create funding strategies together has emerged as a potential opportunity.

For our ER asset hub, emerging insights on price mainly related to issues around funding for third sector organisations. There were also repeated references to the additional stresses caused by the ‘*cost-of-living crisis*’ in interviews. There is an emergent opportunity identified by our community-embedded researcher to focus on ‘*what is ‘free’ – greenspace and wellbeing*’ to contribute to a healthier more resilient society.

In NL, our asset hub stated there were funding pressures to ‘*solve social issues rather than face them*’. There was awareness that ‘*participatory art cannot solve social issues such as poverty and state failure, but it can help us address them*’. The service offer in NLC is often ‘*a clean exchange of cash-for-skills or maybe for experience’* as the statutory organisation ‘*understands how to value people with numbers, but how do we communicate the idea that these projects are valuable beyond numbers? The impact the classes and projects have are valuable but not always easy to capture the monetary value. Some courses in North Lanarkshire are free and some are regular at the point of use but require payment. Councils are having to chase the money to ensure things are financially sustainable which can affect the quality of the courses on offer*’. Transport costs are also prohibitive in NL for people getting in and around to different courses and classes on offer.

Power and price are acutely interlinked in the context of our outlier asset hub, as seen via social and economic inequalities experienced by community members. The sessions and discussions around systems and co-created stories highlight an awareness amongst participants that economic mismanagement, corruption and funding cuts have limited the effectiveness of the systems that currently exist.

During phase 2, our Clacks asset hub formalised their increasingly embedded relationship with the Clacks Council service delivery team, local community services and service users. The hub’s database, which has a comprehensive list of service providers supporting the mapping of service delivery with all actors involved and co-produced with service providers in Clacks, was a key offering.

#### 3.1.6 Purpose

Our ER asset hub surfaced a key theme in interviews related to Integrated Care Systems. While organisations were keen to be part of the hub, there was significant ‘*volunteer fatigue preventing the development of new services and activities*’. Findings also highlight a strong disconnect between the issues affecting communities in ER and the communities’ perceptions about their ability to fix them. This is underpinned by a lack of meaning (or purpose), a lack of faith in ‘the system’ and a sense of powerlessness to change the issues affecting them. The asset hub has established links and partnerships with various health and social care providers all over ER, and identified numerous causes of health disparities and spoken to partners who seek to readdress the balance in these areas. The hub works closely with both providers and service users to offer new ways of looking at health inequalities. The focus of the hub is on cementing these relationships to effect change on the ground by removing reliance on ‘*a broken and fractured public system*’.

**Figure 8:**
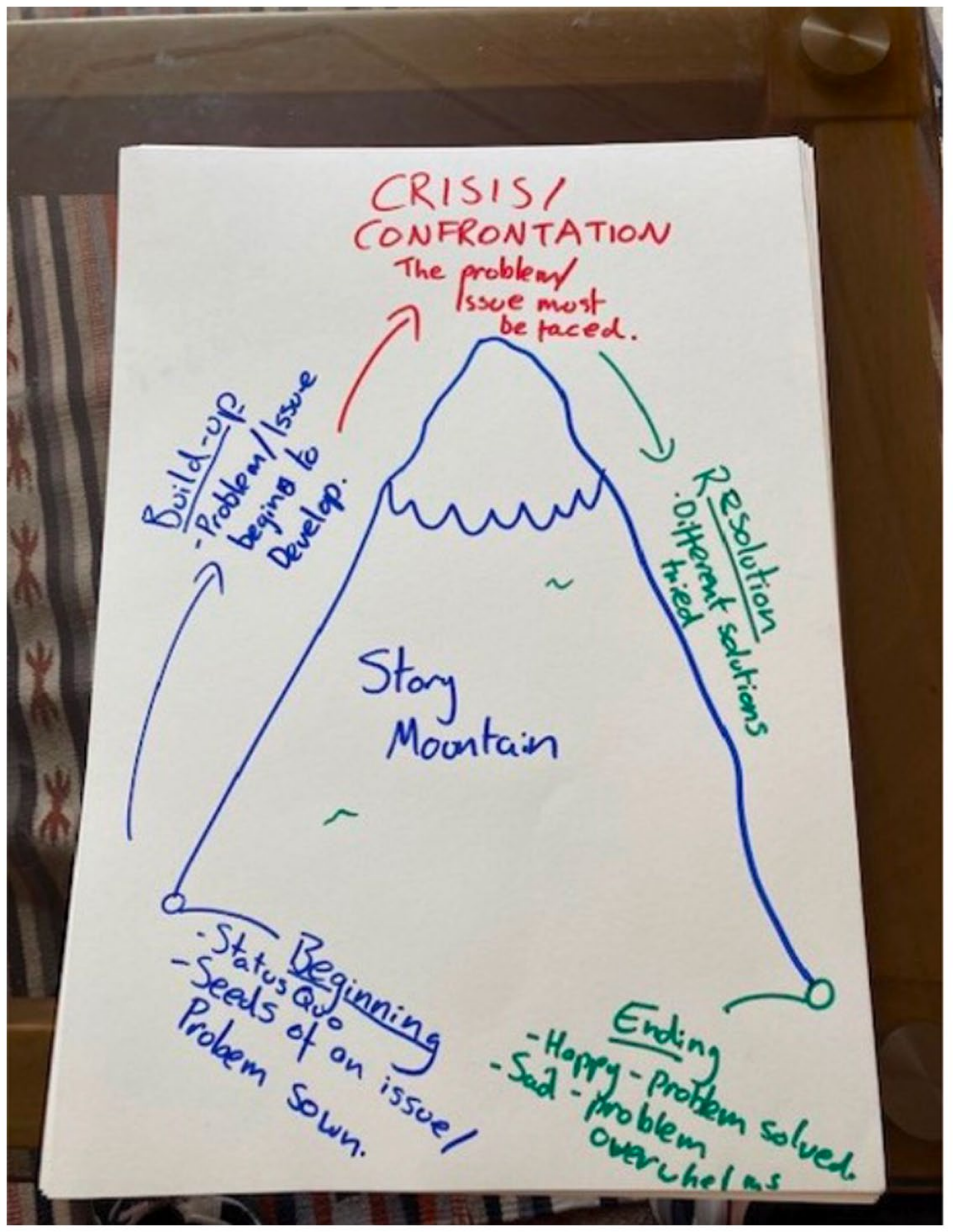
Mapping the recovery journey in North Lanarkshire.

Our NL hub brought together multi-stakeholder agencies, community organisations, creative practitioners, academics, and community members. The work in this funding phase has mainly focused on the Recovery Cafés—led by community organisations with support from the council and REALITIES. This directly tackles health inequalities by addressing complex needs in vulnerable groups, and focuses on integrated care by building connections and community cohesion and opening space for people to ‘recover’ and heal to live healthy, dignified lives (Figure 8).

Through storytelling, our outlier hub highlighted ethical, epistemological and methodological value of participatory and creative methods for exploring health disparities. The data gathered also sheds light on the complexities and interconnections between various elements that affect health outcomes for marginalised communities. It also highlights what is important for the individuals that we worked with—‘*empathic and compassionate systems, as current systems are designed to be void of emotion and treat everyone like a number*’.

### 3.2 Integrating community assets into complex ecosystems

Through further HLS cycles, our critique reflected on power imbalances and inequities in RPP partnerships and offered suggestions for how to nurture healthy ecosystems thereby bridging the gap between research evidence production and public impact.

#### 3.2.1 Overcoming barriers and enabling participation

We acknowledged complexities, barriers and tensions in RPP participation and attempted to overcome these limitations and operational or logistical barriers. These included limited and expensive transport by providing transport to REALITIES sessions; refunding bus tickets; provision of key workers to support people leaving homes; creating inclusive, safe and comfortable workshop spaces in familiar, accessible locations; flexible scheduling; peer-to-peer outreach; creating modes of engagement prioritising community-embedded researchers; and successfully applying for small spin-off grants to further support local communities. We countered digital poverty by providing digital devices through community partners and social enterprises so families could attend creative, digital sessions. When individuals struggled to engage, we connected them to mental health charities in our asset hubs. ‘Pre-pre-employment’ strategies and signposting was offered across our networks to help people build skills to sustain long-term employment. This slow approach addresses psycho-social barriers before preparing them for work to boost self-esteem; tackles numeracy/literacy issues; depression; anxiety; debt, housing and other material barriers. When green and blue spaces were not utilised much by excluded populations leading to disconnection from local ecologies, we designed psycho-social, wellbeing sessions use creativity, nature and social prescribing resources to engage communities with natural environments. When potential political tensions arose across different and within refugee groups, we benefited from co-created sessions with ‘grassroots’ agencies with knowledge of these issues and expertise in sensitive engagement. Emotional support was on offer. Notably, our learning and governance structure is sensitive to potential cultural, linguistic, personal, social, economic and political barriers to participation for communities we work with.

We build on good practices in participatory design, for example Costanza-Chock’s (36) design justice principles, by focusing on healing, empowering and flourishing, what this means for each of the communities we work with in practice and how it will be achieved [will be defined with them]. The framework helped guide and facilitate engagements bespoke to the context, people and purpose of each asset hub (10). Our intention is to continue to make these visible and iteratively respond to tensions by focusing on strategies such as ‘allying’ (engaging in continuous partnerships and facilitating on-going support with community-embedded researchers playing a key role), and ‘resourcing’ (providing people with lived experience with assets they need to design the change they seek), while being supported through allyship and connections to our wider programme networks to influence change (our flat, non-hierarchical learning and governance model spanning the micro, meso, exo, macro systems will support this).

A clear cultural barrier related to this is around (lack of) ‘purpose’, which can be detrimental to meaningful engagement and long-term participation. We acknowledge addressing this requires time and care, as this is about transforming mindsets (how we think) and ‘heartsets’ (how we feel). In phase 3, a key focus is on creating appropriate conditions for genuine co-design across asset hubs, including understanding and framing meaning and purpose—mapping ‘what matters to the people within each asset hub?’, and nurturing agency, hope and trust through co-creating collective visions to achieve outcomes that matter to everyone through dialogue and debate, artefacts, performances, and community action.

#### 3.2.2 Challenges of scaling up, testability and complexity

Measuring change starts with individual(s) and is then related to the system laterally. In other words, change in the individual changes behaviour, which changes the support environment, which changes the behaviour of the support, which leads to more effective ways of relating to each other. REALITIES operates through these different levels and dimensions with findings aligned to these dimensions. At the individual level, stories of people with lived experience bring unique narratives of individual experiences, situations or realities informing the specialist practitioners’ expectations of receiving specific guidance on how to support specific groups. However, the broad element of inequality crosscuts all these dimensions and brings similar issues, feelings and priorities to address.

Researching Evidence-based Alternatives in Living, Imaginative, Traumatised, Integrated, Embodied Systems is like a ‘expandable breathing ball’ making this movement of expansion, where you see specific findings and insights and the next minute you can also make the movement of coming back, becoming a united ball again—a big picture.^2^ In this way, general findings and patterns can be interconnected and presented to address complexity across different organisational levels, but without making ‘generalisations’. We are not dealing with straight lines or even feedback loops, but instead complex relationships and systems that are both practiced and imagined across all of these levels. In that sense, when thinking about ‘generalisability’—or ‘transferability’ of findings—ongoing re-imagining and re-inventing work that is being undertaken in REALITIES will in itself be part of a cumulative ongoing process of creativity and re-imagination that will go well beyond the formal timeframe of the project. Thus, while we envision future practitioners using the REALITIES model in their own work, its inherent flexibility allows for future unique reimagining(s) that are welcomed by us as a consortium. While certain core findings from REALITIES will therefore be transferable, the model is underpinned by local, place-based complexity (which we embrace, alongside local engagement and empowerment) that will drive any future reimagining whether at individual or system level.

Each of our asset hubs work as though they are the only site; staying focused on their own populations; trying to make change at the local level. There will be natural similarities across the sites—and we need the scalability for the findings to have impact beyond local, regional and even national levels—however, we are not seeking to become so ‘meta’ that we lose the granularity of findings for each particular population or asset hub. Indeed, challenges or barriers are key components of the implementation or scaling up process, which will form a core component of our future learning. We will continue to think about the language of barriers and facilitators as we go along, as they can risk a degree of de-contextualisation, which we would also like to retain during the scaling up process. The effectiveness of our RPP as we scale up and build capacity will rely on members of the core team meeting so learning, including policy and practice insights, can happen in ‘real-time’ as REALITIES phase 3 unfolds. Partners will be invited to relevant events and have opportunities for further collaboration as the research progresses.

#### 3.2.3 From siloed learning to transdisciplinary collaboration in practice

Shared values and agreed purpose is central to the functioning of REALITIES—a simple shared achievable aim is set and reviewed at regular intervals. By working with experienced community-embedded researchers and meeting regularly with the core team, the risk of siloed working is minimised and can be brought back at any point through our learning and governance structures that are continually explored, reflected on and created through shared, daily creative and relational practices. Importantly, phase 2 has given us experience of this. Our residential facilitated sharing and mutual learning across disciplinary silos. The title ‘co-investigators’ in this project questions paradigms of power relations traditionally adopted in academia and is in clear alignment with the principles embedded in the proposal—flattening hierarchies and valuing everyone’s contribution.

We are also reflective and critical of our own practices and, though focus is on centring lived experiences, ‘the person/community group’ acts as the point of integration for different conversations and integration of different forms of knowledge systems. We are already implementing a mentorship model being further developed in phase 3 with a mentor-mentee scheme implemented across the multi-site project, particularly for early-career researchers (ECRs) and community-embedded researchers. We will consider flexing these relationships to one of co-mentorship and reverse-mentoring where appropriate, so that senior members of the team can also learn from ECRs. Our strategic approach is to select partner organisations from different facilitated, tailoring their choices to best deliver co-creative participatory action research workshops and REALITIES sessions.

#### 3.2.4 Knowledge exchange and direct impact on communities and frontline practitioners

As evidenced in REALITIES phase 2, frontline practitioners carry a lot of power. They are vehicles to deliver community needs—not the driving force nor influencer of needs seeking to forward their own agenda. In our asset hubs, communities identify barriers and tell us what they need and want to make this better and we utilise creative activity to shine a light on this and gather insights/findings from the grassroots-level upstream. REALITIES offers frontline practitioners direct relationships with researchers and community members to co-create and co-deliver activities. We are simultaneously identifying skills and building capacity of frontline practitioners beyond their specific professional boundary. Tapping into their wider interests and activity preferences often relaxes them and allows the system/user relationship to relax and soften. We know that when community members, practitioners and researchers share perspectives for the same problem they want to address, they felt ‘energised’ – ‘revigorated’ knowing that ‘they are not alone’ in facing their challenges and in making efforts to tackle them. These encounters, permeated by meaningful dialogues and using different ways of communicating, are precious.

Our phase 2 research indicates that knowledge exchange and impact are not expected to occur until the end of the meaning-making process, which is typical in community and action-based research. However, REALITIES brings potential immediate and medium-term impacts considering our extensive network spanning academic, non-academic, and practitioner backgrounds including:

- Increased knowledge of what support is available in communities and awareness of views, expectations and life context of groups we are working with; participation of people with lived experience in creative and relational spaces; and practices promoting critical reflection and creation of collective strategies to overcome problems. This supports people’s wellbeing as it allows a platform to express their feelings, thoughts and identities.
- Knowledge of how the current system is understood from different perspectives within the system (e.g. practitioners, people with lived experience), and power dynamics therein.
- Understanding of key areas of strength/resilience and vulnerability within the current system from those embedded within it.
- Ongoing reflective and reflexive engagement with participants to develop ‘bottom-up’ *in situ* based change during the project life course.
- Inter-relational strengthening of community assets.

#### 3.2.5 Embedding relational ethics and safeguarding

Ethics is a central thread running through REALITIES. The inception of the project is rooted in social justice and based on our understanding of the challenges experienced by people who experience vulnerability when interacting with the elements of health and social care systems that are set up to provide support to them. Thus, from the start, REALITIES has centralised the notion of ethics in the form of social justice. While the outcome is ethically driven, we operationalise our RPP partnerships in a way that attends to the relational; values the experiences of those who find themselves in situations of vulnerability and/or marginalisation; and demonstrates care towards those with whom we work. Our work is underpinned by the concepts of relational vulnerability (37), ethics of care, relational ethics, and an ethics of recognition (38–40). We apply these concepts in a way that is intentional and responsive to the situation of the other.

We maintain close dialogue with organisations, for example those supporting refugee and asylum seekers, that are involved in strategies to mitigate language and cultural barriers. Usually translation of materials to be used in the activities/workshops and a collaboration with a member of the community that speaks English are the most common strategies. Practical considerations include ‘citizen translators’, non-verbal means of sharing, and communication transcending language barriers, for example visual or performative. Our ‘multi-cultural’ programme team also brings awareness and our own lived experience of sensitivities towards diversity and inclusivity. When needed, we work with the Scottish Social Services Council regulatory body or Health and Care Professions Council, which is often the regulator for practitioners in third sector. We also adhere to our institutional safeguarding policies at the University of Edinburgh and, in phase 3, the University of Dundee.

This project has approval from Research Ethics Committee in the School of Health in Social Science, University of Edinburgh. Primarily participants are organisations in which individuals with particular roles participate in HLS PAR cycles focused on making and testing changes in ways of working. The research team does not hold any personal data on these people as this is all held in the organisation.

## 4 Discussion

### 4.1 Methods

Phase 2 confirmed that understanding drivers of inequalities and the role of community assets in reducing differences requires opening up conceptual limitations of health, recovery and what counts as evidence. REALITIES proposes that no singular system of knowledge or perception of reality should be prioritised over another.

Foundational research evidenced REALITIES is able to transcend the challenge for our currently imagined health and social care systems. The medical model of disease shaping who and what is considered to be part of ‘the health system’ has brought benefits to human existence, though key actors within these place-based health and social care systems understand the limitations of this systems-framing for human flourishing. At present, they do not have a way to help reimagine them.

Researching Evidence-based Alternatives in Living, Imaginative, Traumatised, Integrated, Embodied Systems provides exploration and method for this reimagining. A model representing collective pathways producing creative routes for people to get the healthcare they need at the right time of their journeys by co-researching and co-creating with them their outcomes or ‘what, whom, how, and why’—leading to successful connections between individuals with health and social needs and community-based opportunities for health and wellbeing improvement.

We are not saying that doing creative and nature-based activities with communities alone will lead to systems change, nor are we asserting that these initiatives can significantly alter structures in a cause-effect manner. However, our model is resourcing and embedding collaborative networks across Scotland that are adopting a different approach. When these resource networks become interconnected, they can create a power bloc that can challenge dominant cultural norms, ideologies and neo-liberal assumptions around how systems and structures should or can be arranged.

For phase 3, we have an even wider transdisciplinary collective of individuals with lived and felt experience of inequalities working alongside policymakers; local authorities; charities; artists; environmentalists and researchers from policy; health humanities; arts; psychology; human geography; environmental sociology; dentistry; medicine; statistics; economics; counselling; psychotherapy; management; medical anthropology; design and innovation. Through RPP partnerships we will continue to bridge the gap between research evidence production and public impact as we continue to:

- understand what work is needed to enable places to reimagine and build ‘systems’ that create equitable health and wellbeing;
- explore and explain how links between creativity, relationships and nature create healthier and more resilient communities and environments for people in deprived areas;
- support creative, participatory processes, enabling communities to construct shared mental models (systems) using different ways of knowing (epistemologies) and perceiving reality (ontologies);
- combine different ways of knowing, enabling a more complete representation of bio-psycho-social-political factors which create ‘health’ and ways in which these are experienced by marginalised people;
- support communities to construct place-based versions of systems encompassing all aspects of health and wellbeing, and make purposeful changes in the nature of their relationships with each other and their environment; and
- explore the usefulness of ‘standard’ Health Economic evaluation tools to assess Social Return of Investment, working with communities to re-conceptualise and re-define measures of ‘value’ and ‘quality of life’ in relation to human experience.

Perhaps the greatest limitation of this model is our ambition to sustain work in communities and test an alternative approach to measuring change in complex systems while operating alongside and in tandem with these broken systems. To this end, for our model to be translated into ‘reality’ and the implementation process to be afforded, we will need to work tirelessly within and beyond our consortium and continue to build relationships with the institutions that are making application feasible in the ‘real world’. We are encouraged by the attention our research is increasingly receiving from policymakers, third sector and other organisations, though recognise this work takes time and can be swayed by lack of political will and insufficient resourcing. With some partners at risk of burnout and closure, we will need to move forward with care.

## Data availability statement

The original contributions presented in the study are included in the article/supplementary material, further inquiries can be directed to the corresponding author.

## Ethics statement

The studies involving humans were approved by University of Edinburgh, School of Health and Social Sciences Ethics Committee. The studies were conducted in accordance with the local legislation and institutional requirements. Written informed consent for participation in this study was provided by the participants’ legal guardians/next of kin. Written informed consent was obtained from the individual(s) for the publication of any potentially identifiable images or data included in this article.

## Author contributions

MdA – Conceptualization, Data curation, Formal analysis, Funding acquisition, Investigation, Methodology, Project administration, Resources, Supervision, Visualization, Writing – original draft, Writing – review & editing.

## REALITIES consortium

Nicholas Barton-Wines, Rhiannon Bull, Lucy Campbell, Scott Davis, Toby Lowe, Alan Marshall, Aileen Neilson, Mark O’Hare, Sneha Raman, Sam Rowe, Christina Sachpasidi, Candela Sanchez-Rodilla Espeso, Rosie Stenhouse, Leah Soweid.

## Footnotes

^1^Art is Everywhere is a collaborative, place-based knowledge exchange programme aiming to tackle health inequalities through community assets and creative community engagement. It is part of the wider Measuring Humanity project which calls for innovative methods and evidence-gathering through whatever creative or relational form was deemed valid and appropriate for communities, from dance, to theatre, to crafting.

^2^Thanks to Andrea Rodriguez from the University of Dundee, who joined our consortium in phase 3, for this visualisation after observing this movement in a child’s toy.
